# Antibody Profiling in Naïve and Semi-immune Individuals Experimentally Challenged with *Plasmodium vivax* Sporozoites

**DOI:** 10.1371/journal.pntd.0004563

**Published:** 2016-03-25

**Authors:** Myriam Arévalo-Herrera, Mary Lopez-Perez, Emmanuel Dotsey, Aarti Jain, Kelly Rubiano, Philip L. Felgner, D. Huw Davies, Sócrates Herrera

**Affiliations:** 1 Malaria Vaccine and Drug Development Center (MVDC), Cali, Colombia; 2 Faculty of Health, Universidad del Valle, Cali, Colombia; 3 Caucaseco Scientific Research Center, Cali, Colombia; 4 Department of Medicine, University of California Irvine, Irvine, California, United States of America; Johns Hopkins Bloomberg School of Public Health, UNITED STATES

## Abstract

**Background:**

Acquisition of malaria immunity in low transmission areas usually occurs after relatively few exposures to the parasite. A recent *Plasmodium vivax* experimental challenge trial in malaria naïve and semi-immune volunteers from Colombia showed that all naïve individuals developed malaria symptoms, whereas semi-immune subjects were asymptomatic or displayed attenuated symptoms. Sera from these individuals were analyzed by protein microarray to identify antibodies associated with clinical protection.

**Methodology/Principal Findings:**

Serum samples from naïve (n = 7) and semi-immune (n = 9) volunteers exposed to *P*. *vivax* sporozoite-infected mosquito bites were probed against a custom protein microarray displaying 515 *P*. *vivax* antigens. The array revealed higher serological responses in semi-immune individuals before the challenge, although malaria naïve individuals also had pre-existing antibodies, which were higher in Colombians than US adults (control group). In both experimental groups the response to the *P*. *vivax* challenge peaked at day 45 and returned to near baseline at day 145. Additional analysis indicated that semi-immune volunteers without fever displayed a lower response to the challenge, but recognized new antigens afterwards.

**Conclusion:**

Clinical protection against experimental challenge in volunteers with previous *P*. *vivax* exposure was associated with elevated pre-existing antibodies, an attenuated serological response to the challenge and reactivity to new antigens.

## Author Summary

Malaria remains an important public health problem worldwide, with 13.8 million cases caused by *Plasmodium vivax*, a parasite species that predominates in South-East Asia and the American continent. Despite the epidemiological importance of this species, studies of the immune response and their potential for vaccine development are limited. Here we use a high-throughput technique (protein microarray) to identify antibodies in serum from malaria naïve and semi-immune Colombian volunteers experimentally infected with *P*. *vivax*. We show a higher response in semi-immune individuals before the challenge. Meanwhile, at day 45 after infection, both groups had the highest antibody response to several *P*. *vivax* proteins. Additional analysis indicated that semi-immune volunteers without fever recognized new antigens, which may represent promising targets for vaccine development. Taken together, these findings represent a significant step forward in the understanding of the humoral immune response to *P*. *vivax* malaria infection, particularly the extent of immune priming upon a first parasite encounter.

## Introduction

Malaria remains an important public health problem worldwide, affecting mainly developing countries in Africa, Asia and Latin America. The World Health Organization estimated that 214 million cases of malaria occurred worldwide in 2015 [1]. Of these cases, 13.8 million cases were calculated to be caused by *Plasmodium vivax*, a parasite species that predominates in South-East Asia and the American continent where it accounts for more than 50% of malaria cases [1].

In areas of high malaria transmission, individuals continuously exposed to *Plasmodium* develop partial protection against severe symptoms at an early age and a significant number of asymptomatic infections are recorded [2]. This clinical protection is mediated by both innate and acquired mechanisms that are not well understood [2–4]. Under conditions of hypo- or meso-endemic transmission, both clinical and subclinical infections are seen in all age groups and, despite the lower frequency of malaria exposure, significant protection against the disease is induced [5]. A high prevalence of uncomplicated and asymptomatic *P*. *vivax* and *P*. *falciparum* malaria infections are reported in both hyperendemic and unstable malaria transmission regions, indicating that a significant level of clinical immunity is induced by repeated exposure to the parasite [2, 6–9].

Specific antibodies against *P*. *vivax* and *P*. *falciparum* proteins have been reported to be associated with clinical immunity [2, 4, 10]. However, only a few antigens have been made available through traditional cloning methods or peptide synthesis. Sequenced *P*. *vivax* and *P*. *falciparum* malaria parasite genomes, along with high-throughput proteomic techniques and bioinformatics are powerful tools currently available for systematic analyses of humoral immune responses associated with naturally and experimentally induced malaria. These analyses provide a better understanding of malaria parasite-host interaction, disease pathogenesis, host immune response and the identification of potential vaccine candidate antigens [11–13]. Despite the epidemiological importance of *P*. *vivax*, the immune mechanisms and their potential for vaccine development have been studied less than in *P*. *falciparum*. Currently, only two parasite antigens, *Pv*CSP and *Pvs*25 have been assessed in early clinical development [14–16] as vaccine candidates, although several others are in preclinical development [17–19].

In recent years, the Malaria Vaccine and Drug Development Center (MVDC) in Cali (Colombia) has standardized a safe and reproducible method for *P*. *vivax* sporozoite challenge by *Anopheles albimanus* mosquito bites [20, 21]. This method enables the evaluation of the protective efficacy of *P*. *vivax* vaccine candidates under controlled conditions, accelerating their clinical development both by facilitating efficacy studies and antigen discovery. In this context, a challenge study was recently conducted in malaria-naïve and semi-immune volunteers, who were exposed to controlled *P*. *vivax* infected mosquito bites [22]. Although all study subjects became parasitemic at the same time point after *P*. *vivax* challenge, all naïve volunteers developed symptomatic infections while semi-immune volunteers had either only mild symptoms or no symptoms. Antibody responses against two immune-dominant *P*. *vivax* antigens, *Pv*CSP and *Pv*MSP1, showed no differences in the frequency of responders, although naïve volunteers exhibited significantly higher antibody responses to these antigens [22]. In order to fully characterize the natural protective antibody responses and to better understand the responses induced by *P*. *vivax* infection in both study groups, a protein microarray displaying 515 *P*. *vivax* antigens was probed with serum samples from these volunteers.

## Methods

### Ethics statement

This trial was conducted according to ICH E-6 Guidelines for Good Clinical Practices [23] and the protocol was approved by Institutional Review Boards (IRB) of the MVDC and Centro Médico Imbanaco in Cali. Written informed consent was obtained from each volunteer at enrollment. The clinical trial was registered on clinicaltrials.gov, registry number NCT01585077. The protocol for this trial is available as supporting information (S1 Protocol).

### Study participants and sample collection

Blood samples were collected from malaria-naïve (n = 7) and semi-immune (n = 9) adult volunteers that participated in a clinical trial carried out at the MVDC [22]. Malaria-naïve volunteers were recruited in Cali (a non-endemic city) and declared not having suffered malaria and lack of previous malaria exposure was ascertained by negative indirect fluorescent antibody test (IFAT). Semi-immune volunteers were recruited in Buenaventura (malaria-endemic area) and previous malaria exposure was confirmed by clinical history as well as by the presence of antibodies against *P*. *vivax* blood stages and sporozoites detected by IFAT.

**Fig 1:**
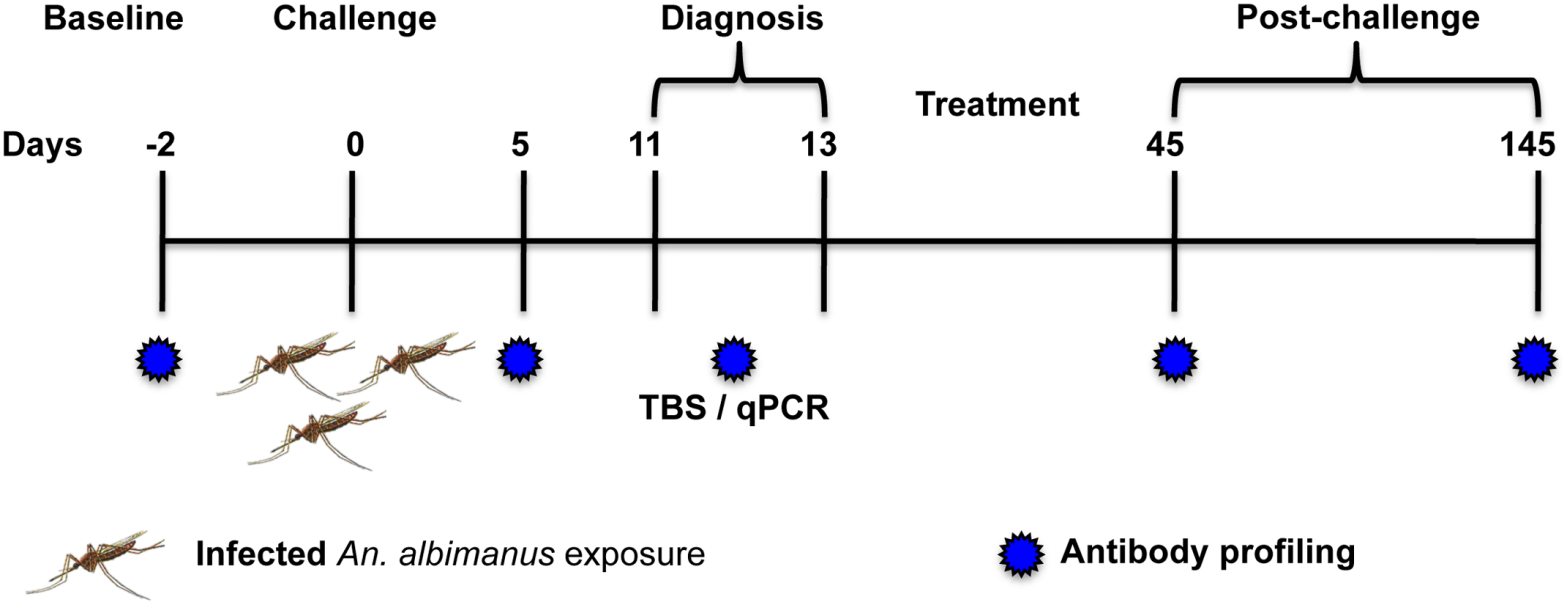
Schematic representation of the study. Naïve (n = 7) and semi-immune (n = 9) volunteers were challenged by exposure to the bites of 2–4 *P*. *vivax* infected mosquitoes. Patent blood-stage parasitemia was detected by thick blood smear (TBS) and confirmed by real time qPCR on days 11 to 13 post-challenge. All volunteers were treated orally with chloroquine and primaquine and followed-up until day 145 after challenge.

All volunteers were challenged by exposure to bites of two to four mosquitoes previously fed with *P*. *vivax*-infected blood obtained from a malaria patient (field strain) as reported before [22]. Volunteers were followed-up for malaria signs and symptoms and were treated orally with curative doses of chloroquine (25 mg/kg) split in three doses and primaquine (0.5 mg/kg daily) for 14 days, as recommended by the official Colombian guidelines, as soon as parasites were detected by microscopy [24]. Serum samples were collected before the challenge (baseline) and five, 11–13 (here day 11), 45 and 145 days after the challenge (Fig 1). Detailed information about demographic characteristics of the study participants, challenge infective dose, pre-patent period, parasite density after challenge, and clinical and laboratory evaluations was previously reported [22].

### Protein microarray

A custom protein microarray (Pf/Pv500) displaying 515 *P*. *vivax (Pv)* and 500 *P*. *falciparum* proteins expressed on pre-erythrocytic and asexual parasite blood stages and printed as *in vitro* transcription/translation (IVTT) system was purchased from Antigen Discovery Inc., (Irvine, CA). Arrays content was down-selected from the *Pv* 4,506-protein microarray based on seroreactivity as detailed previously [25]. Although volunteers’ samples were hybridized to the whole array, data for *P*. *vivax* antigens only are presented in this paper. Microarray information is publicly available on the NCBI Gene Expression Omnibus (http://www.ncbi.nlm.nih.gov/geo/) and is accessible through accession number GPL18316. Annotation of proteins presented in this study follows gene accession numbers published on PlasmoDB (www.plasmodb.org). Of 515 *P*. *vivax* features on the array, 444 mapped to unique *P*. *vivax* proteins, of which the majority (247; 56%) were classified as hypothetical proteins or hypothetical conserved proteins. Each array contained 24 negative “IVTT-control” reaction spots lacking plasmid template expression, which provide a donor-specific ‘background’ signal that was used to normalize data between individuals.

For probing, serum samples were diluted 1:100 in protein array blocking buffer (Maine Manufacturing, Sanford, ME) supplemented with *E*. *coli* lysate (GenScript, Piscataway, NJ) to reach a final concentration of 10mg/ml, and pre-incubated at room temperature (RT) for 30 min. Concurrently, arrays were rehydrated in blocking buffer (without lysate) for 30 min. Arrays were probed with pre-incubated serum samples overnight at 4°C with gentle agitation, and then washed at RT five times with TBS-0.05% Tween 20 (T-TBS), followed by incubation with biotin-conjugated goat anti-human IgG (Jackson ImmunoResearch, West Grove, PA) diluted 1:200 in blocking buffer for one hour at RT. After incubation with secondary antibodies, arrays were washed three times in T-TBS and bound IgG was visualized using streptavidin-conjugated SureLight P-3 (Columbia Biosciences, Frederick, MD) diluted 1:1000 in blocking buffer for 45 min at RT in the dark. Arrays were washed three times with T-TBS, and once with water. Chips were air-dried by brief centrifugation and scanned in a GenePix 4200AL laser scanner (Molecular Devices, Sunnyvale, CA). All samples in this study were probed at the same time on the same batch of arrays.

### Data analysis

Analysis of the protein microarray data was accomplished following our previously published computational methods [3, 11]. Briefly, microarray spot intensities (median fluorescence intensity, MFI) were quantified using ScanArray Express software (Perkin Elmer, Waltham, MA) and IVTT spot intensities were normalized by subtraction of the sample-specific median of the IVTT control spots. Antigens were considered seroreactive if the spot intensity of an individual (or the average for a group of individuals) was greater than a cutoff defined as the average plus two standard deviations of the reactivity to all *P*. *vivax* antigens in a US control population. Antibody breadth was used as defined for *P*. *falciparum* [26] as the number of seroreactive antigens per individual or group. Venn diagrams of group antibody breadths were produced using the BioVenn web application (http://www.cmbi.ru.nl/cdd/biovenn/index.php) [27]. Statistical analyses were performed on data normalized by dividing the IVTT signal by the sample-specific median of the IVTT control spots division (fold-over control, FOC) and taking the base 2 logarithm of the ratio (Log2 FOC). Differentially reactive proteins between both groups were determined using Wilcoxon rank-sum test, and those with Log2 FOC > 1 considered seropositive (Prism v6.0, GraphPad Software Inc., La Jolla CA). A p value < 0.05 was considered statistically significant.

## Results

### Study population characteristics

Volunteers were adults aged between 19 and 38 years. Briefly, all volunteers developed infections, which were confirmed by microscopy and RT-qPCR, with similar median parasitemias between naïve and semi-immune volunteers (36 parasites/μL; IQR 9.0–98.8 *vs* 55 parasites/μL; IQR 29.5–163.5; p = 0.288). All naïve volunteers presented with classical malaria signs and symptoms, while semi-immune volunteers displayed minor or no symptoms on the day of diagnosis [22].

### Characterization of *P*. *vivax* reactive targets before challenge

**Fig 2:**
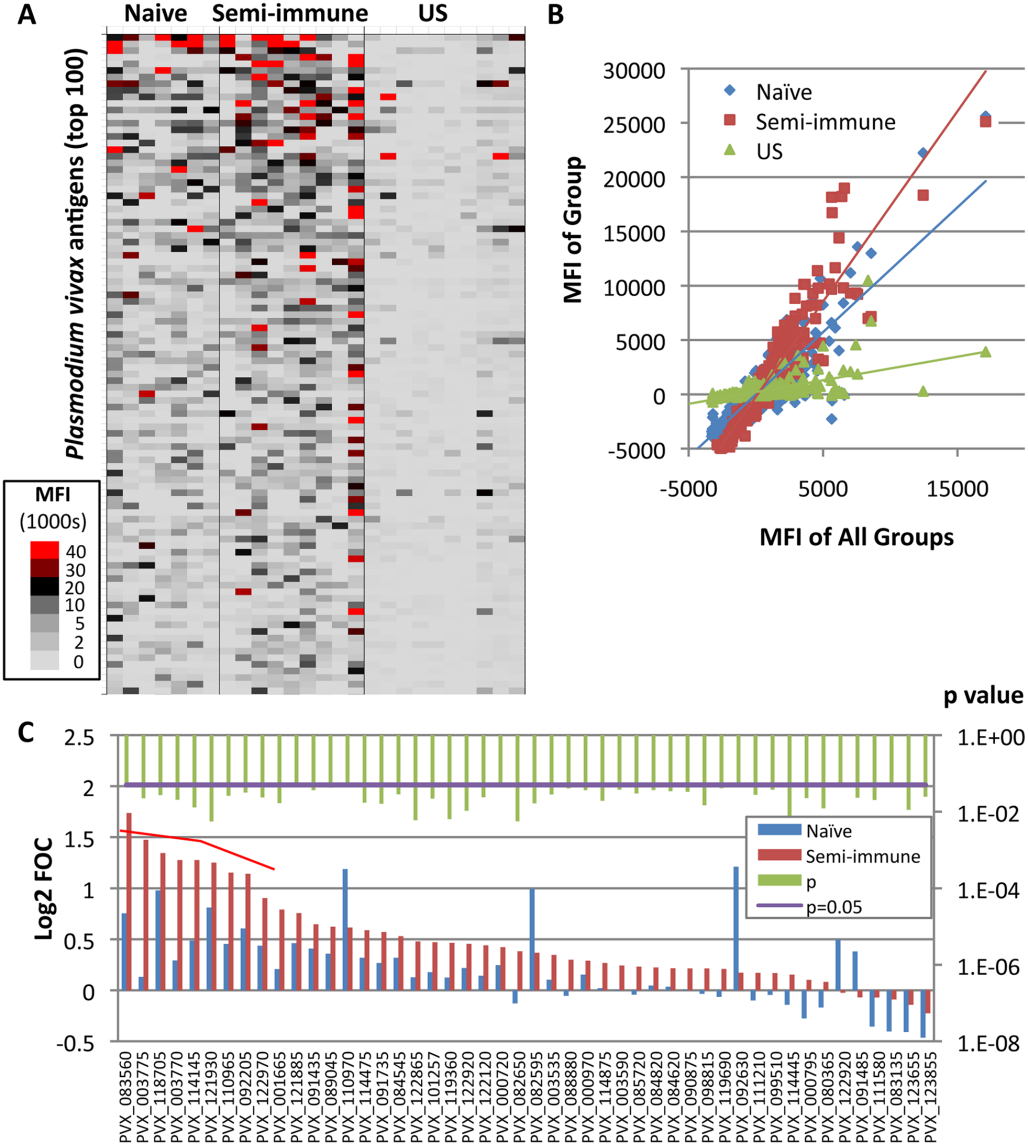
Antibody profiling in Colombian individuals before *P*. *vivax* challenge. *Plasmodium vivax* protein arrays were probed with serum samples collected before challenge (day 0) and at four time-points afterwards, as shown in the schematic in Fig 1. **A**. Heat map showing serological profiles on day 0 prior to challenge for each Colombian naïve and semi-immune individual, and US controls for comparison. Raw signal intensities for each IVTT spot have been subtracted from the sample-specific median of background (IVTT control) spots, and the adjusted signal intensity represented by a color according to the key. Only the top 100 antigens are shown, ranked by average adjusted signals of both Colombian groups. **B**. Scatter plot of individual antigens, in which the average signal of each group (y-axis) is plotted against the average of all three groups (x-axis); the slope of the regression line is proportional to the overall breadth and intensity of the profile in each group. Each point represents the median fluorescence intensity (MFI) for all individuals examined in the particular group to a particular antigen. **C**. Bar chart of normalized array data (Log2 FOC) at 1:200 serum dilution. Only antigens with significant reactivity difference (p<0.05) between naïve and semi-immune volunteers are shown (raw p-values; green bars). Of all the significant antigens, nine were considered seropositive (i.e., using Log2 FOC >1 as the cutoff; red bracket); these are shown in Table 1.

Fig 2A shows a heat map of ‘subtracted’ array data (IVTT values minus sample-specific IVTT controls signals) for each naïve and semi-immune individual, and for US controls. The analysis revealed higher reactive responses in semi-immune than naïve individuals before the challenge, and both groups’ responses were higher than those in the US controls. This differential reactivity is seen more clearly from the slopes of the linear regression lines when average signal intensities from each group are plotted against the average of all three groups (Fig 2B). The steeper slope of the semi-immune individuals relative to the naïve individuals confirms an overall higher reactivity in this group. The breadth of the baseline antibody profile, defined as the sum of reactive *P*. *vivax* antigens per individual, ranged from three to 71 reactive antigens for naïve individuals and three to 89 for semi-immune individuals. While the average group antibody breadth was broader for the semi-immune group (179 antigens) in comparison to naïve volunteers (113 antigens), both groups shared reactivity for 98 of the antigens. Only a single seropositive antigen (PVX_003775, MSP4) was significant when naïve and semi-immune groups were compared (p < 0.05). To test whether this small number of differences was influenced by serum dilution, arrays were probed at 1:200 and 1:400 dilutions. A dilution of 1:200 yielded eight differentially reactive antigens with a Log2 FOC >1 (Fig 2C and Table 1). The majority of these were merozoite surface antigens, consistent with previous exposure to blood-stage parasites, with only one non-annotated antigen represented. At 1:400 dilution the number of reactive antigens fell to only two (PVX_003775, MSP4 and PVX_003770, MSP5) indicating that the 1:200 dilution is optimal to maximize differences seen between Colombian naïve and semi-immune individuals. In conclusion, IgG antibodies to several *P*. *vivax* antigens were more elevated in semi-immune Colombian individuals than naïve Colombian individuals, although both groups had elevated antibodies compared to naïve US controls.

**Table 1:** The PlasmoDB gene ID and description of the top antigens that discriminate between naïve and semi-immune individuals at baseline.

ORF PlasmoDB ID	Product description	Exon	Log 2 FOC normalized data^a^		p value^b^
			Naïve	Semi-immune	
PVX_083560	Hypothetical protein, conserved	2 of 2	0.754	1.738	0.047
PVX_003775	Merozoite surface protein 4 (MSP4) putative	2 of 2	0.131	1.474	0.023
PVX_118705	Hypothetical protein, conserved	1 of 1	0.979	1.344	0.027
PVX_003770	Merozoite surface protein 5 (MSP 5)	1 of 2	0.292	1.276	0.021
PVX_114145	Merozoite surface protein 10 (MSP10)	1 of 1	0.486	1.276	0.013
PVX_121930	*Plasmodium* exported protein, unknown function	2 of 2	0.811	1.251	0.006
PVX_110965	Merozoite surface protein 3 (MSP3)	1 of 1	0.453	1.153	0.026
PVX_092205	Ubiquitin domain containing protein	1 of 1	0.605	1.141	0.032

^*a*^FOC, fold-over control. Values > 1 (i.e., two-fold over the IVTT controls spots) were considered seropositive.

^*b*^p value using Wilcoxon Rank-Sum Test.

### Antibody reactivity induced after *P*. *vivax* challenge

**Fig 3:**
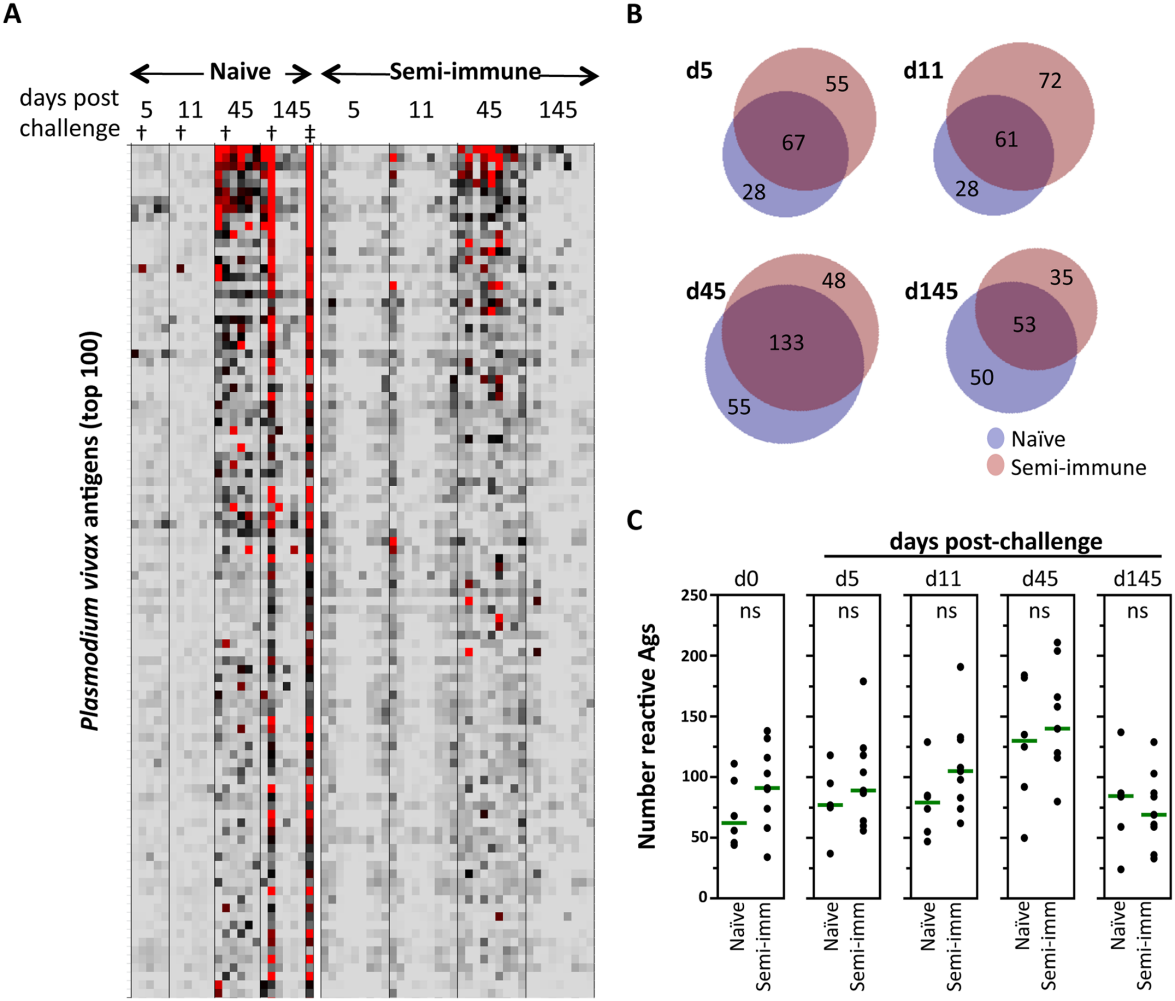
Antibody reactivity after challenge. **A**. Heat map of array data for all four post-challenge time points. Data were normalized by subtraction of IVTT controls, as described in Fig 2, and then subtracted from day 0 values to reveal more clearly the change in the profile due to challenge. The profile in one atypical naïve individual who presented with a new *P*. *vivax* infections on day 130 (‡), indicated by the dagger (†) in each time point is also shown. **B**. Venn diagrams of specific and shared antigens at each of the post-challenge time points. An antigen was defined as reactive if the average per group > avg + 2SD of the US controls; data for the atypical naïve individual were removed from this analysis. **C**. Dot plots showing numbers of reactive antigens for naïve and semi-immune volunteers at each post-challenge time point; data for the atypical naïve individual were removed for this analysis. Green lines = medians; ns = not significant (Wilcoxon Rank-Sum test).

To normalize differences in background reactivity seen between both study groups and to reveal only the signals induced in response to the *P*. *vivax* challenge, pre-existing background reactivity at baseline (day 0) for each antigen was subtracted from the later time points data. In the semi-immune volunteers, the reactivity after challenge corresponded to a boosting of antibodies already present at baseline as well as appearance of new ones. At day five, reactivity of a few proteins was significantly higher in semi-immune than in naïve volunteers: serine-repeat antigen 5 (SERA5; PVX_003830) and three hypothetical proteins with unknown function (PVX_094690, PVX_084120, PVX_113590). However, at diagnosis day (day 11) the antibody response to *P*. *vivax* remained similarly low in both groups (Fig 3A–3C).

Notably, reactivity rose abruptly on day 45 in both groups, followed by a decline to near baseline by day 145 (Fig 3A). The profile in one naïve volunteer (indicated by † in Fig 3A) who presented with a new *P*. *vivax* infection on day 130 (indicated by ‡ in Fig 3A) did not decline by the final time point. Indeed, the profile remained strong at a follow-up time point of 145 days. Since the serological dynamic of this individual was different to the others in the group, these data were removed from subsequent analyses. The expansion of the profile as measured by the group antibody breadth (Fig 3B), was marginally more rapid in the semi-immune group, although at response peak (day 45) the breadths were roughly equivalent in both groups (naive = 188; semi-immune = 181; total reactivity = 236). Both group profiles declined thereafter with roughly equivalent breadths at day 145 (naïve = 103; semi-immune = 88; total reactivity = 138). The response dynamics are shown by the dot plots of antibody breadth (Fig 3C). These data were not subtracted from baseline signals to more clearly show the challenge-induced increase in the breadth relative to the pre-challenge baseline.

To determine how long remain the antibodies elicited against *P*. *vivax* antigens without parasite re-exposure, the individuals were followed-up for 145 days. On this day several antigens were identified as significant when naïve and semi-immune were compared, although only six were considered seropositive (Log2 FOC > 1); two of them were higher in semi-immune volunteers (SERA5 and a hypothetical protein, PVX_094690). In contrast, naïve volunteers had higher response to MSP1, MSP8, ETRAMP and a hypothetical protein with unknown function (PVX_083560; S1 Table).

### Antibody profile associated with *P*. *vivax* malaria clinical protection

**Fig 4:**
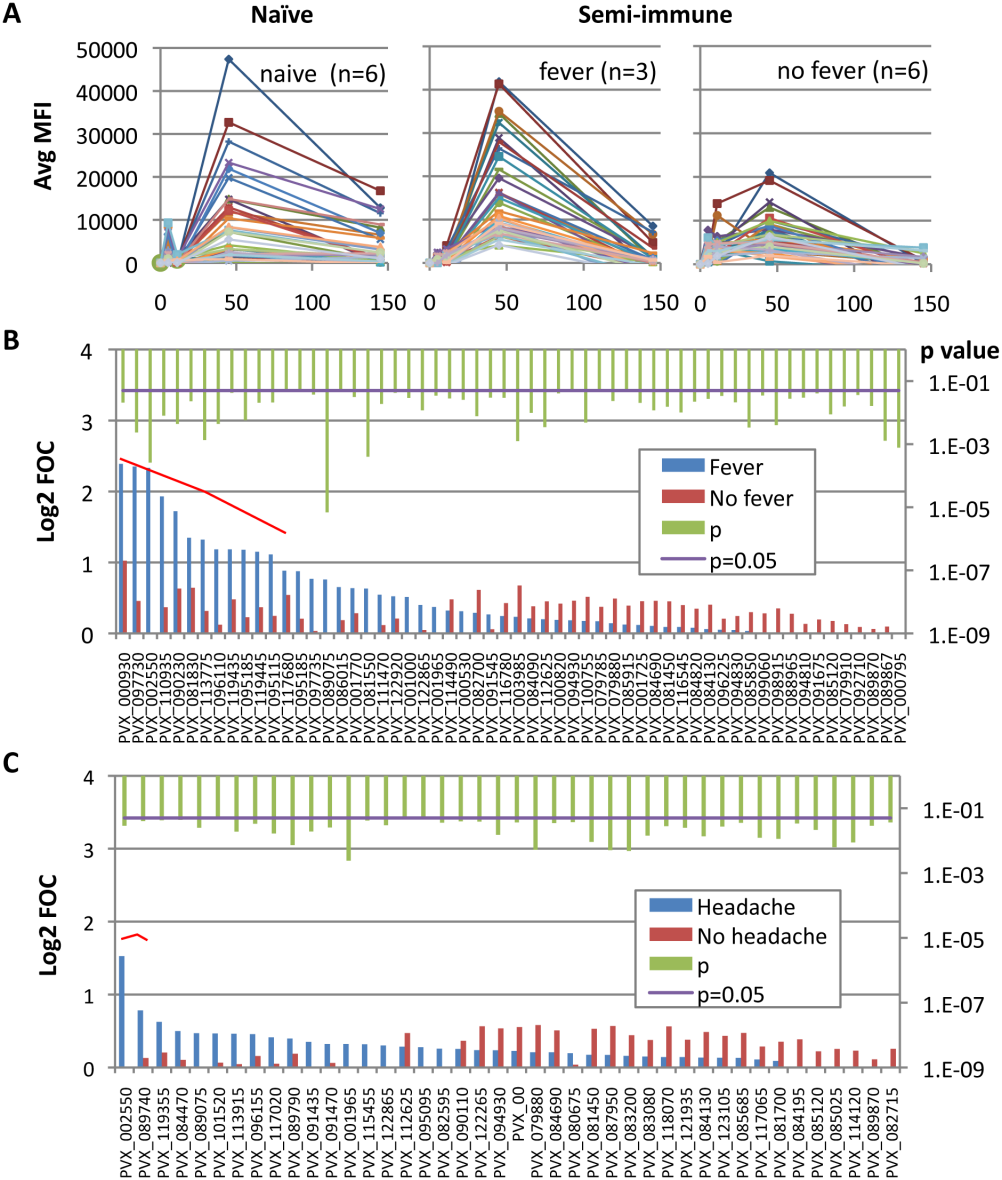
Antibody profile associated with clinical protection. **A**. Kinetics of antibody response against *P*. *vivax* antigens. Semi-immune volunteers were segregated into those that developed fever and those who did not. Average of median fluorescence intensity (MFI) is shown. **B**-**C**. Bar graph of normalized array data (Log2 FOC) for top individual antigens in semi-immune volunteers at day 45 segregated by fever (blue bars) or no fever (red bars) (**B)** and headache (blue bars) *vs*. no headache (red bars) **(C)**. P values in Log scale (green bars) using the Wilcoxon Rank-Sum Test are shown with the purple line representing the significance threshold (p = 0.05). Red bracket indicates seropositive antigens. Only antigens with significant reactivity (p<0.05) are shown.

As described [22], naïve individuals all developed classical malaria symptoms such as headache, fever, nausea, chills, and malaise associated with *P*. *vivax* challenge at the time of parasite patency. In contrast, semi-immune volunteers reported either no symptoms or only minor symptoms associated with the *P*. *vivax* appearance in blood; only 33% presented fever (body temperature ≥ 38°C). Therefore, semi-immune volunteers were segregated into those that developed symptoms (or “non-protected”) or did not develop symptoms (or “protected”). The group means of the top 40 individual antigens subtracted from baseline signals showed that those semi-immune individuals that developed fever after challenge had a robust (naïve-like) response that peaked on day 45, while the asymptomatic individuals showed an attenuated response at this time (Fig 4A). Both returned to near baseline by day 145. Segregation using headache as a symptom was also analyzed with similar results (S1 Fig).

Comparison of the protected and unprotected semi-immune profiles on day 45 identified several antigens as significant when data were segregated by fever (Fig 4B and Table 2), although only 12 were considered seropositive (Log2 FOC >1; indicated by the bracket in Fig 4B). Interestingly, all of them were higher in semi-immune volunteers with fever. In semi-immune individuals segregated by headache, several antigens were significant, although only one (PVX_002550; conserved hypothetical) was considered seropositive (Fig 4C).

**Table 2:** Top reactive antigens at day 45 after challenge that discriminate between semi-immune individuals with fever or without fever.

ORF PlasmoDB ID	Product description	Exon	Log 2 FOC normalized data^a^		p value^b^
			Avg Fever	Avg No Fever	
PVX_000930	sexual stage antigen s16, putative	1 of 1	2.389	1.024	0.021
PVX_097730	hypothetical protein, conserved	1 of 1	2.352	0.458	0.002
PVX_002550	hypothetical protein, conserved	2 of 3 S2	2.332	-0.032	0.000
PVX_110935	hypothetical protein, conserved	1 of 1	1.930	0.368	0.008
PVX_090230	early transcribed membrane protein (ETRAMP)	1 of 2	1.721	0.632	0.004
PVX_081830	hypothetical protein	2 of 2	1.347	0.643	0.023
PVX_113775	6-cysteine protein (P12)	1 of 1	1.322	0.316	0.001
PVX_096110	hypothetical protein, conserved	1 of 5 S2	1.185	0.123	0.004
PVX_119435	activator of Hsp90 ATPase, putative (AHA1)	5 of 5	1.184	0.480	0.042
PVX_095185	hypothetical protein, conserved	1 of 2	1.179	0.229	0.006
PVX_119445	FAD dependent glycerol-3-phosphate dehydrogenase, putative	1 of 1	1.151	0.368	0.021
PVX_095115	D123 (regulator of eIF2), putative	1 of 3	1.113	0.247	0.021

^*a*^FOC, fold-over control. Values > 1 (i.e., two-fold over the IVTT controls spots) were considered seropositive.

^*b*^p value using Wilcoxon Rank-Sum Test.

To test the hypothesis that several antigens recognized in semi-immune individuals at the peak of the response after challenge were “new” antibodies absent from the baseline profile, as opposed to boosted from antibodies present at baseline, statistical comparison between profiles at day 0 (baseline) *vs*. day 45 (peak) was performed. Those that were protected or non-protected (using fever as the symptom) were analyzed separately. Volunteers without fever developed antibodies to 13 new antigens, including three members of the MSP family (one, seven and 10) and three hypothetical proteins, whereas individuals with fever had reactivity to 16 new antigens. However, antibodies to only five new antigens were shared by both groups, all of them with higher reactivity in volunteers with fever (Table 3). These data suggests that only one *P*. *vivax* infection is enough to induce antibody response against new antigens.

**Table 3:** New antigens at day 45 after challenge in semi-immune individuals with fever or without fever^a^.

ORF PlasmoDB ID	Product description	Log 2 FOC normalized data^b^		p value^c^
		Avg Fever	Avg No Fever	
PVX_002550	hypothetical protein, conserved	2.407	< 1	0.000
PVX_097625	Merozoite Surface Protein 8 (MSP8)	2.303	< 1	0.002
PVX_097730	hypothetical protein, conserved	1.983	< 1	0.000
PVX_113245	Cyclin dependent protein kinase, predicted	1.921	< 1	0.001
PVX_092995	Tryptophan rich antigen (Pvfama)	1.872	< 1	0.003
PVX_094965	hypothetical protein, conserved	1.315	< 1	0.047
PVX_110965	Merozoite surface protein 3 (MSP3)	1.242	< 1	0.049
PVX_095185	hypothetical protein, conserved	1.146	< 1	0.020
PVX_092070	hypothetical protein, conserved	1.110	< 1	0.044
PVX_113775	membrane protein pf12 precursor, putative	1.038	< 1	0.009
PVX_117150	26S proteasome subunit, putative	1.014	< 1	0.005
PVX_083560	hypothetical protein, conserved	< 1	2.381	0.000
PVX_099980	Merozoite surface protein 1 (MSP1)	< 1	2.154	0.000
PVX_082680	Merozoite surface protein 7 (MSP7), putative	< 1	1.407	0.041
PVX_114145	Merozoite surface protein 10 (MSP10)	< 1	1.372	0.031
PVX_003840	Serine repeat antigen 3 (SERA3)	< 1	1.286	0.005
PVX_121930	hypothetical protein, conserved	< 1	1.243	0.007
PVX_000995	Transmission blocking target antigen Pfs230, putative	< 1	1.108	0.027
PVX_084985	hypothetical protein, conserved	< 1	1.094	0.010
PVX_000930	sexual stage antigen s16, putative	2.828	1.652	0.000
PVX_118705	Hypothetical protein, conserved	2.687	1.878	0.000
PVX_081830	hypothetical protein	2.243	1.578	0.026
PVX_090230	early transcribed membrane protein (ETRAMP)	1.994	1.263	0.029
PVX_115450	membrane associated histidine rich protein (MAHRP1)	1.783	1.190	0.010

^*a*^New antigens at day 45 that were absent from the baseline profile (day0).

^*b*^FOC, fold-over control. Values > 1 (i.e., two-fold over the IVTT controls spots) were considered seropositive.

^*c*^p value using Wilcoxon Rank-Sum Test between day 0 and day 45.

## Discussion

This study revealed that individuals who were semi-immune to *P*. *vivax* had pre-existing antibodies that although present at low levels were associated with clinical protection to *P*. *vivax* sporozoite experimental challenge [22]. As expected, semi-immune volunteers showed higher reactivity than naïve individuals to several *P*. *vivax* antigens before challenge. Moreover, exposure to a presumably low dose of viable sporozoites inoculated by the bites of only 2–4 mosquitoes was enough to induce a robust antibody response in malaria-naïve volunteers as well as to trigger antibody responses to new antigens in semi-immune volunteers (Table 3). Another valuable observation was that a proportion of the anti-*P*. *vivax* antibodies were short-lived as 138 of the 236 antigens (>40%) recognized by day 45 had disappeared by day 145 after challenge. The rapid decay of a subset of antibodies indirectly indicated that semi-immune volunteers had not had recent exposure to the parasites, because several of these antigens were not recognized at pre-challenge time.

Before challenge, the Colombian malaria-naïve individuals had significantly higher serological reactivity than the US controls, despite being residents of a non-endemic malaria area. They were confirmed as seronegative against *P*. *vivax* blood stages and sporozoites using IFAT. Although infections or experience with protozoa were not studied here, the reactivity observed in Colombian naïve individuals might be due to other pathogens such as *Cryptosporidium parvum* or others highly prevalent in Colombia [28]; *C*. *parvum* shows homology with several *Plasmodium* proteins [29]. Nevertheless, this serological reactivity did not appear to have played a role in protection as all naïve volunteers developed malaria-related symptoms and patent parasitemia at the expected time [20–22]. The higher reactivity of the semi-immune volunteers to several antigens before challenge as compared to naïve volunteers indicates that in endemic regions, even with low transmission intensity, they develop and maintain *P*. *vivax* specific antibodies to a broad number of antigens even after a few previous malaria episodes (2–5 episodes). However, the degree of immunity conferred by these pre-existing antibodies was not enough to modify the pre-patent period or parasitemia at diagnosis day, although it was highly effective in controlling malaria symptoms.

Interestingly, in the subgroup of semi-immune volunteers that developed fever or headache, as in the naïve, the antibody response to challenge was more vigorous than that in asymptomatic volunteers who displayed an attenuated antibody response. This is consistent with findings from *P*. *falciparum* vaccination studies in humans where protected individuals did not mount a significant antibody response to challenge, whereas unprotected subjects responded to challenge by elevated signals to many blood stage antigens [11, 30]. Although in those studies *Pf*CSP was recognized by both the protected and unprotected subgroups, protected individuals had a significantly higher magnitude of response [11, 30].

At day 45 volunteers with fever showed a significantly higher response to *P*. *vivax* antigens such as MSP3, MSP4, MSP5 and MSP10. However, reactivity to *Pv*MSP1 and *Pv*CSP, two established vaccine candidates [14, 31], was not different between volunteers with and without fever, as previously seen for the same sera using a recombinant *Pv*MSP1 fragment (r200L) and synthetic *Pv*CSP construct by ELISA [22]. These results partially contrast with those of epidemiological studies on *P*. *vivax* where an association between sera reactivity to MSP1, MSP3 and MSP9 proteins and clinical protection has been reported [10, 32–34]. The higher reactivity to the CSP in *P*. *falciparum* studies [30, 35] is most likely due to the multiple immunization doses, while here only a few mosquito bites were allowed, with possibly low sporozoite density sufficient to induce infection once and a detectable antibody levels against a high number of other *P*. *vivax* antigens in all volunteers.

In summary, the antibody profiles that developed in humans after experimental exposure to *P*. *vivax* sporozoites were defined. It was shown that a single infection was enough to induce detectable specific antibodies in malaria naïve volunteers and to boost the antibodies elicited by natural exposure to malaria in semi-immune individuals. Comparison between semi-immune volunteers segregated by fever showed that those protected had an attenuated serological response after challenge, but also had reactivity to new antigens, which may represent promising targets for vaccine development. Taken together, these findings represent a significant step forward in the understanding of the humoral immune response to *P*. *vivax* malaria infection, particularly the extent of priming upon a first parasite encounter.

## Supporting Information

S1 Table Significant reactive antigens at day 145 after challenge. (DOC)

S1 Fig Kinetics of antibody response to *P*. *vivax* antigens in semi-immune volunteers segregated by headache. The volunteers were segregated into those that reported headache and those who did not. Average of median fluorescence intensity (MFI) of top 40 individual antigens is shown.
(TIF)

S1 Protocol Comparison of the susceptibility of naïve and pre-immune volunteers to the Infectious challenge with viable *Plasmodium vivax* sporozoites. (DOCX)
