# Supplementary material for: Antibody Profiling in Naïve and Semi-immune Individuals Experimentally Challenged with Plasmodium vivax Sporozoites
Source: PLoS Negl Trop Dis. 2016 Mar 25;10(3):e0004563. doi: 10.1371/journal.pntd.0004563 (PMC4807786; doi:10.1371/journal.pntd.0004563)
Supplement: S1 Table — (DOC) [file pntd.0004563.s001.doc]

# S1 Table. Significant reactive antigens at day 145 after challenge

| **ORF**  **PlasmoDB ID** | **Product description** | **Log 2 FOC normalized data*^a^*** | | **p value*^b^*** |
| --- | --- | --- | --- | --- |
|  |  | **Naïve** | **Semi-immune** |  |
| PVX_094690 | hypothetical protein, conserved | -1.943 | 1.281 | 0.000 |
| PVX_003830 | Serine repeat antigen 5 (SERA5) | -2.204 | 1.139 | 0.000 |
| PVX_083560 | hypothetical protein, conserved | 1.320 | 0.539 | 0.001 |
| PVX_089375 | hypothetical protein, conserved | -0.558 | 0.495 | 0.000 |
| PVX_080365 | Eukaryotic translation initiation factor 3 subunit 9, putative | -0.113 | 0.378 | 0.043 |
| PVX_123510 | S4, putative | -0.171 | 0.325 | 0.041 |
| PVX_099980 | Merozoite Surface Protein 1 (MSP1) | 1.022 | 0.314 | 0.004 |
| PVX_097625 | Merozoite Surface Protein 8 (MSP8) | 1.380 | 0.265 | 0.000 |
| PVX_001770 | hypothetical protein | 0.817 | 0.209 | 0.012 |
| PVX_114255 | hypothetical protein, conserved | 0.653 | 0.149 | 0.038 |
| PVX_003565 | early transcribed membrane protein (ETRAMP) | 1.066 | 0.143 | 0.001 |
| PVX_083135 | aspartate carbamoyltransferase, putative | 0.645 | 0.125 | 0.032 |
| PVX_113825 | hypothetical protein, conserved | 0.638 | 0.016 | 0.010 |
| PVX_097735 | hypothetical protein | 0.648 | -0.036 | 0.005 |
| PVX_084625 | P-type ATPase4, putative | 0.469 | -0.047 | 0.034 |
| PVX_121980 | 40S ribosomal protein S7, putative | -0.795 | -0.050 | 0.002 |
| PVX_090230 | early transcribed membrane protein (ETRAMP) | 0.782 | -0.070 | 0.001 |
| PVX_085550 | hypothetical protein, conserved | 0.531 | -0.074 | 0.013 |
| PVX_090215 | hypothetical membrane protein, conserved | 0.368 | -0.153 | 0.032 |
| PVX_121885 | cytoadherence linked asexual protein, CLAG, putative | 0.774 | -0.194 | 0.001 |
| PVX_089695 | hypothetical protein, conserved | 0.371 | -0.292 | 0.006 |

*^a^*FOC, fold-over control. Values > 1 (i.e., two-fold over the IVTT controls spots) were considered seropositive (shown in gray). *^b^*p value using Wilcoxon Rank-Sum Test
