# Supplementary figures and images for: Antibody Profiling in Naïve and Semi-immune Individuals Experimentally Challenged with Plasmodium vivax Sporozoites
Source: PLoS Negl Trop Dis. 2016 Mar 25;10(3):e0004563. doi: 10.1371/journal.pntd.0004563 (PMC4807786; doi:10.1371/journal.pntd.0004563)

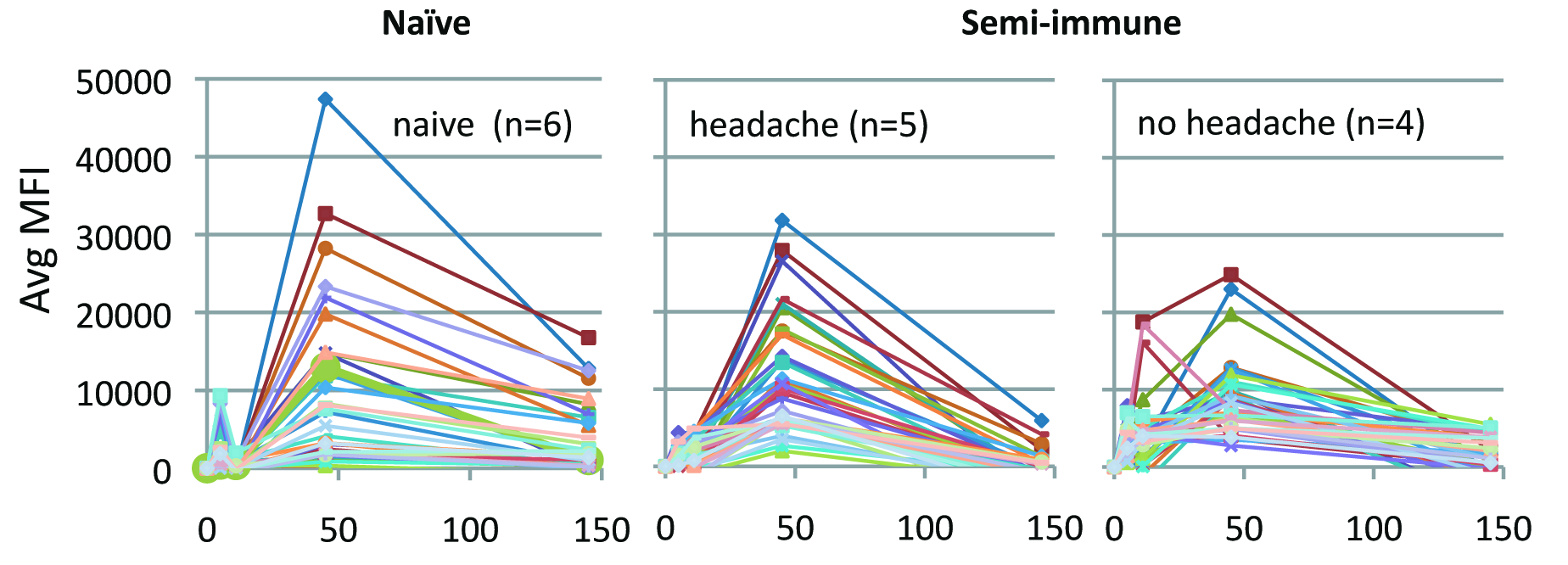

Supplement: S1 Fig — The volunteers were segregated into those that reported headache and those who did not. Average of median fluorescence intensity (MFI) of top 40 individual antigens is shown. (TIF) [file pntd.0004563.s002.tif]
